# Megahertz-rate shock-wave distortion cancellation via phase conjugate digital in-line holography

**DOI:** 10.1038/s41467-020-14868-y

**Published:** 2020-02-28

**Authors:** Yi Chen Mazumdar, Michael E. Smyser, Jeffery D. Heyborne, Mikhail N. Slipchenko, Daniel R. Guildenbecher

**Affiliations:** 1 Sandia National Laboratories, P.O. Box 5800, Albuquerque, NM 87185 USA; 2 School of Mechanical Engineering, Georgia Institute of Technology, Atlanta, GA 30332 USA; 3 School of Mechanical Engineering, Purdue University, West Lafayette, IN 47907 USA

**Keywords:** Nonlinear optics, Imaging and sensing, Nonlinear optics, Imaging techniques

## Abstract

Holography is a powerful tool for three-dimensional imaging. However, in explosive, supersonic, hypersonic, cavitating, or ionizing environments, shock-waves and density gradients impart phase distortions that obscure objects in the field-of-view. Capturing time-resolved information in these environments also requires ultra-high-speed acquisition. To reduce phase distortions and increase imaging rates, we introduce an ultra-high-speed phase conjugate digital in-line holography (PCDIH) technique. In this concept, a coherent beam passes through the shock-wave distortion, reflects off a phase conjugate mirror, and propagates back through the shock-wave, thereby minimizing imaging distortions from phase delays. By implementing the method using a pulse-burst laser setup at up to 5 million-frames-per-second, time-resolved holograms of ultra-fast events are now possible. This technique is applied for holographic imaging through laser-spark plasma-generated shock-waves and to enable three-dimensional tracking of explosively generated hypersonic fragments. Simulations further advance our understanding of physical processes and experiments demonstrate ultra-high-speed PCDIH techniques for capturing dynamics.

## Abstract (web summary)

Shock-waves in explosive, supersonic or ionizing environments impart phase distortions to holographic imaging. Here, the authors report an ultra-high-speed phase conjugate digital in-line holography technique where a laser passes through the shock-wave and is reflected back through the phase distortion, thus correcting phase delays.

## Introduction

Coherent imaging methods like digital in-line holography (DIH)^1,2^ are essential for three-dimensional (3D) object tracking along a single line-of-sight. Traditional DIH techniques have been successfully applied in various systems, from high-speed multi-phase flows^3,4^ to combustion environments^5–7^. Supersonic, hypersonic, and explosive environments, however, create challenges for coherent imaging methods like DIH due to phase distortions generated by shock-waves. Density gradients from bubbles in liquid flows^8^, gas ionization^9,10^, or even strong temperature gradients^11^ also produce phase distortions that inhibit accurate coherent imaging. In previous film-based holographic-imaging experiments in cavitating environments^12^, for example, phase distortions prevented quantitative measurements. Additionally, phenomena which contain shock-wave distortions are also inherently highly dynamic, requiring megahertz-rate time-resolved tracking to determine shock-wave velocities, fragment velocities, and particle impact effects over time. Some of these physical processes are also not repeatable from test to test, making it difficult to obtain time-resolved information via experimental repetition. In order to apply DIH for time-resolved, three-dimensional tracking in explosive and hypersonic environments with shock-wave distortions, new techniques are needed.

To address some of these challenges, a phase conjugate digital in-line holography (PCDIH) technique can be used. This method operates by first passing a forward beam of coherent light through the shock-wave phase distortion. The light then enters a phase conjugate mirror to produce a return beam with the conjugate phase. By passing the return beam back through the phase distortion, the phase delays are corrected but holograms of absorptive or opaque objects are not affected. The concept of phase conjugate mirrors has been used in a variety of applications, such as for removing imaging distortions^13–17^, focusing through turbid media^18,19^, reflecting seed-amplified pulses while dumping background amplified spontaneous emission^20–24^, and eliminating modal dispersion^25^. Phase conjugation techniques have also been implemented using film^26–28^ and spatial light modulators^29–34^. These two methods, however, are not compatible with the ultra-high-speed imaging requirements. Methods for phase conjugation that utilize degenerate four-wave-mixing, on the other hand, can operate at the necessary speeds.

In Guildenbecher et al.^35^, single-shot shock-wave distortion correction was demonstrated via PCDIH for the first time. However, this previous work was limited to 10–20 Hz repetition rates due to power limitations for continuous operation with picosecond and nanosecond lasers^35,36^. As a result, only a single snapshot could be captured per experiment and the fundamental high-speed dynamics could not be measured. With recent developments in pulse-burst laser technologies^21,37,38^, innovations in laser diagnostics at high repetition rates^39–42^, and advancements in ultra-high-speed camera designs^43^, it is now feasible to develop new techniques for ultra-high-speed PCDIH. Flashlamp-pumped pulse-burst lasers, however, have notoriously poor beam quality making them appear to be unacceptable for both coherent imaging and four-wave-mixing. Additionally, as the repetition rate increases, the energy per pulse decreases significantly, making pulse-burst lasers appear to be inappropriate for ultra-high-speed nonlinear four-wave-mixing processes.

**Fig. 1:**
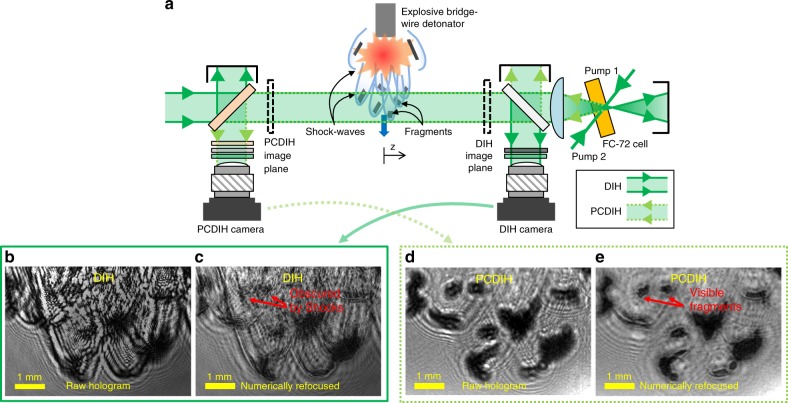
Phase conjugate digital in-line holography measurements. **a** Simplified setup for simultaneous DIH and PCDIH for imaging of explosively generated hypersonic fragments is illustrated. Raw and numerically refocused DIH images **b** and **c** are shown with the raw and numerically refocused PCDIH images **d** and **e** of hypersonic fragments surrounded by shock-wave distortions. These numerically refocused images taken with a picosecond laser show how PCDIH successfully cancels phase distortions that obscure objects.

In this work, we introduce a megahertz-rate PCDIH technique capable of producing quantitative video-rate holograms in explosive, supersonic, and hypersonic environments, as illustrated in Fig. 1a. By operating at up to 2–5 MHz (or million-frames-per-second) using a pulse-burst laser, we demonstrate more than five orders of magnitude increase in speed over previous work^35,36^. Incidentally, the simultaneously obtained 5 MHz DIH data also represents the fastest digital holograms recorded to-date^44–48^. To achieve this, we made several innovations to improve pulse energy, beam quality, and optical setup efficiency. Hence, this work is also the first to combine a flashlamp-pumped pulse-burst laser with coherent imaging and the first to use a nanosecond pulse-burst laser for megahertz-rate four-wave-mixing. To better understand the physical mechanisms, we further outline numerical simulations and experiments that explain the distortion-correcting characteristics of PCDIH, as well as the source of previously unexplained^35^ interference patterns visible in PCDIH images. Finally, experiments are conducted to study the dynamics of laser-spark plasma-generated shock-waves and explosively generated hypersonic fragments. This work illustrates unique capabilities for coherent imaging in both dynamic and stochastic environments. Thus, these techniques can be applied in a variety of other areas including spray systems for cooling or combustion, explosive environments, index gradients in plasmas, and multiphase fluid flows.

## Results

### Principle of operation

Digital in-line holography^1,2,49–51^ operates by passing an incident laser beam *E*_r_(*x*, *y*) = *A*_r_(*x*, *y*)e^*ikz*−*iωt*^ over an object field. Each object has a transmission function *t*(*x*, *y*) = e^−*a*(*x*, *y*)+*iϕ*(*x*, *y*)^ with different absorption *a*(*x*, *y*) and phase delay *ϕ*(*x*, *y*) properties. Downstream of the object, the electric field propagation along the *z*-axis follows the Fresnel–Kirchhoff diffraction function^49^, and can be approximated as

1 $${E}_{{\rm{t}}}(x,y,z)=({E}_{{\rm{r}}}(x,y)\cdot t(x,y))\otimes {g}_{{\rm{f}}}(x,y,z),$$

where  ⊗ is the convolution operator and *g*_f_ = *FT*^−1^(*G*) is the inverse Fourier transform of the diffraction kernel $G={{\mathrm{{e}}}}^{(2\pi iz/\lambda )\sqrt{1-{(\lambda u)}^{2}-{(\lambda v)}^{2}}}$ defined in the *u*–*v* spatial frequency coordinate system. An example of the interference pattern *h*(*x*, *y*) =∣*E*_t_(*x*, *y*, *z*_h_)∣^2^ collected downstream at the holography image plane *z*_h_ is illustrated in Fig. 1b. Here, a raw hologram of explosively generated hypersonic fragments surrounded by shock-wave distortions is shown. This raw hologram is numerically refocused using,

2 $${E}_{{\rm{h}}}(x,y,z)=\left[h(x,y){E}_{{\rm{r}}}^{* }(x,y)\right]\otimes {g}_{{\rm{b}}}(x,y,z),$$

where *E*_h_ is the numerically reconstructed complex amplitude, ${E}_{{\rm{r}}}^{* }(x,y)$ is the planar conjugate reference wave, and *g*_b_ = *FT*^−1^(G^*^) is the inverse Fourier transform for the complex conjugate of *G*. The numerical refocusing algorithms described by these equations are implemented using HoloSAND. Note that DIH is typically collected out-of-focus and later numerically refocused to determine 3D positions. The refocused image at any depth *z* is visualized using the amplitude *A*_h_ = ∣*E*_h_∣ and the *z*-location of each object is then found using a focus metric that minimizes amplitude and maximizes edge sharpness^52^. Through this technique, DIH can be used to localize amplitude objects in three dimensions.

The addition of an unknown phase object, however, can severely distort image reconstruction making it difficult to segment object edges from *A*_h_ as illustrated in Fig. 1c. For a simplified example^53^, a phase object with delay *ϕ*_s_(*x*, *y*) alters the total beam such that ${E}_{{\rm{t}}}^{\prime}(x,y,z)={E}_{{\rm{t}}}(x,y,z){{\mathrm{{e}}}}^{i{\phi }_{{\rm{s}}}(x,y)}$. If the beam reflects off of an ordinary mirror with reflectivity *R*_m_, the electric field after the mirror and after passing through the phase delay a second time are

3 $${E}_{{\rm{t}}}^{{\prime\prime} }(x,y,z)={R}_{{\rm{m}}}{E}_{{\rm{t}}}(x,y,z){{\mathrm{{e}}}}^{i{\phi }_{{\rm{s}}}(x,y)},$$

4 $${E}_{{\rm{t}}}^{{\prime\prime\prime} }(x,y,z)={R}_{{\rm{m}}}{E}_{{\rm{t}}}(x,y,z){{\mathrm{{e}}}}^{2i{\phi }_{{\rm{s}}}(x,y)}.$$

Here, the final electric field gains twice the phase delay. However, if a phase conjugate mirror with reflectivity *R*_pc_ is used, the electric field after reflection off of the phase conjugate mirror and after passing back through the phase delay are

5 $${E}_{{\rm{t}}}^{{\prime\prime} }(x,y,z)={R}_{{\rm{pc}}}{E}_{{\rm{t}}}(x,y,z){{\mathrm{{e}}}}^{-i{\phi }_{{\rm{s}}}(x,y)},$$

6 $${E}_{{\rm{t}}}^{{\prime\prime\prime} }(x,y,z)={R}_{{\rm{pc}}}{E}_{{\rm{t}}}(x,y,z).$$

Therefore, the phase distortions picked up from the forward pass are corrected on the second pass. The raw PCDIH image collected from this technique (Fig. 1d) can then be numerically refocused (Fig. 1e) showing clearer object edges that are otherwise obscured by shock-wave distortions.

**Fig. 2:**
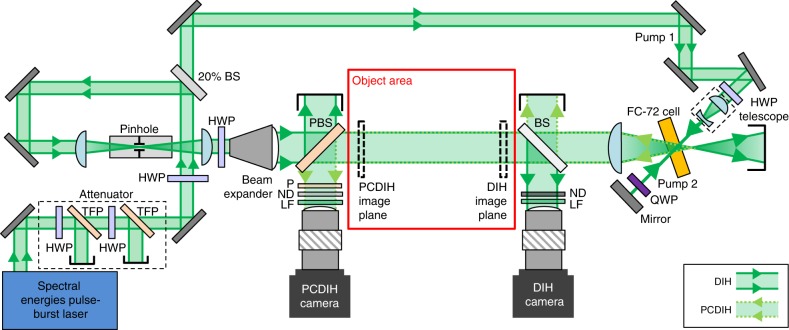
Experimental configuration. Simultaneous DIH and PCDIH using an ultra-high-speed pulse-burst laser is illustrated (HWP—half wave plate, QWP—quarter wave plate, TFP—thin-film polarizer, BS—beam splitter, PBS—polarizing beam splitter, P—polarizer, ND—neutral density filter, LF—laserline filter with 0.2 nm full width at half maximum).

To create the phase conjugate mirror for ultra-high-speed applications, a degenerate four-wave-mixing^53,54^ topology with a double-pass pump beam is implemented. A custom-modified Spectral Energies QuasiModo pulse-burst laser^48^ operating at 532 nm is utilized. Compared with previous work using this laser^39^, a higher-energy pulsed seed laser, software modifications, beam expansions, and reduced pulse durations were needed to enable 5 MHz operation without damage to optics. In Fig. 2, the laser is then split into an imaging beam and a single pump beam. The imaging beam is then used to generate DIH and PCDIH holograms using long distance microscopes and ultra-high-speed Shimadzu HPV-X2 cameras.

To create the phase conjugate beam, the imaging beam with amplitude *A*_3_ is first directed into a phase conjugate mirror fluid^55^ cell. The pump 1 beam (amplitude *A*_1_) is also delayed and directed into the phase conjugate mirror. By reflecting off of a normal mirror, the pump 2 beam (amplitude *A*_2_) traveling in the opposite direction is subsequently created from the pump 1 beam^36,56^. When these three beams arrive in the nonlinear medium simultaneously, a fourth phase conjugate beam with amplitude *A*_4_ and conjugate phase is generated. Additional derivation details for this process are described in Supplementary Note 1 and additional details for this setup are listed in the “Methods” section. The reflectivity of the phase conjugate mirror,

7 $${R}_{{\rm{pc}}}=\frac{| {A}_{4}{| }^{2}}{| {A}_{3}{| }^{2}}={\tan }^{2}\left(\left|\frac{\omega }{2c{n}_{0}(\omega )}{\chi }_{{\rm{e}}}^{(3)}{A}_{1}{A}_{2}\right|l\right),$$

determines the intensity of the reflected beam, where ${\chi }_{{\rm{e}}}^{(3)}$ is the third-order nonlinear susceptibility of phase conjugate mirror fluid, *n*_0_(*ω*) is the fluid index of refraction, and *l* is the interaction length of the laser beams in the cell. Note that the reflectivity scales as ${\tan }^{2}$ of the pump beam amplitude. In contrast to previous work^35,36^, the current design achieves a higher collection efficiency by double-passing the pump beam, adding polarization control elements, and optimizing beam size. These innovations enable ultra-high-speed operation with the pulse-burst laser for coherent imaging and four-wave-mixing.

## Experimental measurements

**Fig. 3:**
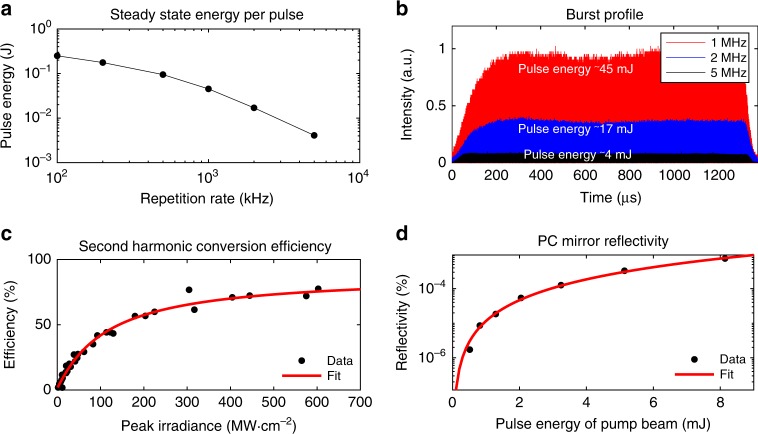
Ultra-high-speed PCDIH system characterization. **a** Average pulse energy at steady state and **b** burst profiles at 532 nm are shown. **c** The second harmonic conversion efficiency from 1064 to 532 nm with logistic fit and **d** the phase conjugate mirror reflectivity as a function of pump energy with a tangent-squared fit to the analytical expression in Eq. (7) are also illustrated. Source data are provided in the Source Data file.

Phase conjugate signals created via four-wave mixing with a nanosecond laser require high 532 nm pulse energies. Generating high pulse energies becomes more challenging as the repetition rate increases into the megahertz regime. For the topology of this laser, the energy per burst is fixed by the burst duration and other characteristics of the amplification stages. Therefore, as the repetition rate increases, the energy per pulse drops, as shown in Fig. 3a and b. When the energy per pulse drops, the conversion efficiency from 1064 nm laser light to 532 nm also decreases, as described in Fig. 3c. Finally, nonlinear conversion in the phase conjugate mirror via the four-wave-mixing process also acts to further reduce the signal strength, as illustrated in Fig. 3d. Here, the measured reflectivity scales as a function of ${\tan }^{2}$, which matches the functional form of Eq. (7). These reflectivity values are relatively low when compared to picosecond implementations^35^ due to differences in peak intensities of picosecond versus nanosecond beams with similar energies per pulse. All of these factors contribute to the challenges associated with implementing PCDIH at ultra-high speed. However, results with the optical arrangement described in this work illustrate that there is sufficient energy for making phase conjugate measurements at up to 5 MHz.

**Fig. 4:**
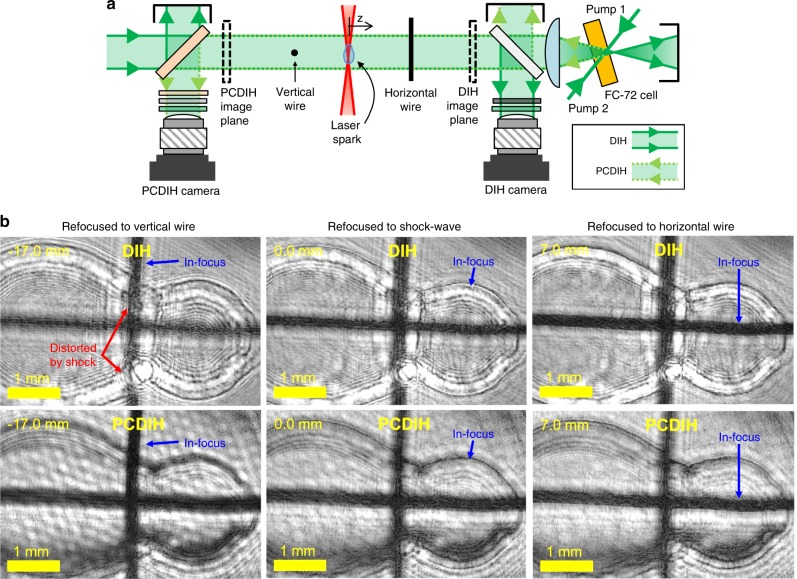
Numerically refocused holograms of a laser-spark plasma-generated shock-wave. **a** Simplified setup and **b** refocused holograms are illustrated. A single hologram from each camera is refocused to the focal plane of the vertical wire (*z* = −17 mm), laser spark (*z* = 0 mm), and horizontal wire (*z* = 7 mm). Note that the overall image brightness was increased to improve visibility and that the pixels are not saturated. Supplementary Movie 1 associated with this figure illustrates numerical refocusing.

To demonstrate ultra-high-speed PCDIH and DIH for capturing dynamics and to better understand the underlying physics that produce different imaging artifacts, a simple setup containing opaque objects and shock-wave distortions was created. As illustrated in Fig. 4a, a focused nanosecond laser is used to generate shock-waves via laser-induced breakdown of the air. A pair of crossed 0.2 mm diameter wires is placed in the object area on either side of a laser spark. The DIH and PCDIH imaging planes are located ~30 mm from the laser spark. Figure 4b shows holograms numerically refocused to the vertical wire, shock-wave edge, and horizontal wire. In this data, the DIH images show bright-fringes generated by the phase-delay through the shock-wave, strong shock-wave phase distortions on the vertical wire, and significantly weaker distortions on the horizontal wire. For the in-line configuration, the difference between the vertical and horizontal wire distortions is due mostly to object and shock-wave order. The corresponding PCDIH images, however, show significantly reduced distortions and no bright fringes from the phase-delay, illustrating the distortion-correcting capabilities of this technique and the unique artifacts generated by the shock-wave. Supplementary Movie 1 demonstrates numerical refocusing for DIH and PCDIH, each from a single hologram.

**Fig. 5:**
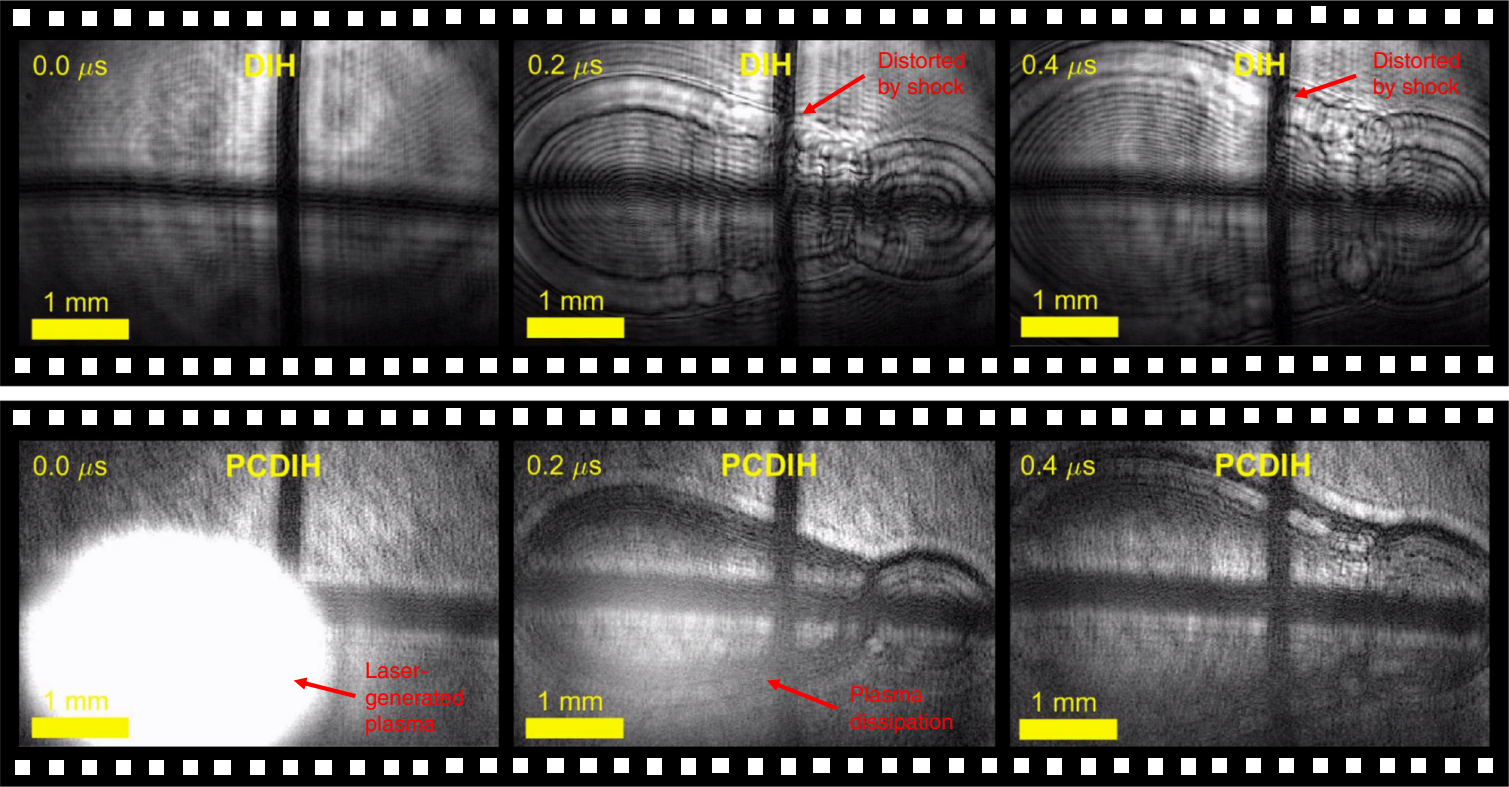
Laser-spark plasma-generated shock-wave videos collected at 5 MHz. The dynamics of shock-wave expansion over time are illustrated from data collected at ultra-high imaging rates. For this particular experiment, the plasma emission overwhelms the first frame of the PCDIH signal. Holograms captured at 500 kHz and 5 MHz are illustrated in Supplementary Movie 2.

Supplementary Movie 2 shows videos captured at both 500 kHz and 5 MHz. When operating at 500 kHz, only a single frame of the shock-wave edges can be captured per test in the field-of-view. In the 5 MHz video and in Fig. 5, the frame rate is sufficiently fast to capture plasma emission and shock-wave expansion over several frames. As the repetition rate is increased, the PCDIH signal-to-noise ratio decreases and the reduction in signal on the PCDIH camera allows plasma emission to overwhelm the phase conjugate signal in the first frame. Depending on signal levels, scattering of the incident DIH laser signal due to ionization and dissociation of the gas^9,10^ could also potentially overwhelm the signal at the PCDIH camera.

**Fig. 6:**
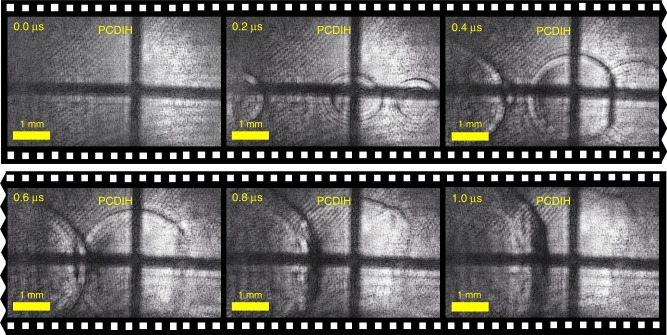
Shock–wave interactions collected at 5 MHz with PCDIH. In this example produced with the same laser-spark energy as Fig. 5, the plasma does not overwhelm the PCDIH signal. Additionally, shock–wave interactions can be observed.

The laser-spark plasma-generation process can be stochastic, and from shot to shot the plasma kernel shapes are not repeatable, especially at early times. Figure 6 shows a second example of PCDIH collected at 5 MHz with the same laser-spark energy. In this case, the light emission from plasma generation is weaker and does not overwhelm the PCDIH hologram. From these time-resolved images, it is possible to track the growth rate of the shock-wave edges and visualize the interaction of multiple shock-waves as they create Mach stems. These results show that time-resolved ultra-high-speed measurements, rather than experimental repetition with previously available techniques, are required for capturing dynamics.

**Fig. 7:**
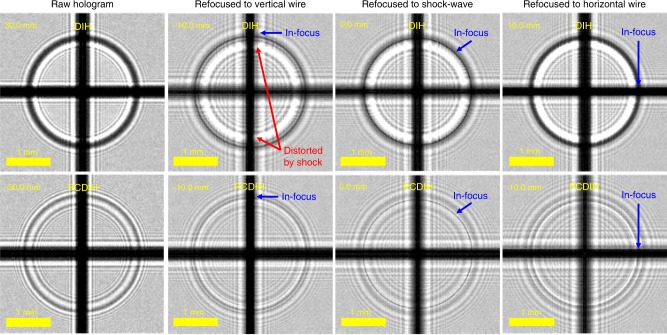
DIH and PCDIH simulations of laser-spark plasma-generated shock-waves. These simulations show a vertical wire (*z* = −10 mm), shock-wave plane (*z* = 0 mm), and horizontal wire (*z* = 10 mm) captured from the DIH image plane (*z* = 30 mm) and PCDIH image plane (*z* = −30 mm). The phase conjugate mirror is located at *z* = 510 mm. For this simulation, the spherical shock-wave has a constant shocked-gas density, shock-wave motion during laser light time-of-flight, and refraction effects. From left to right, the simulated raw recorded hologram, hologram refocused to the vertical wire, hologram refocused to the shock-wave edge, and hologram refocused to the horizontal wire are shown. This can be qualitatively compared with experimental results in Fig. 4.

From the basic theory^53^ presented in Eqs. (5) and (6), pure phase-objects should not be visible in PCDIH. Interestingly, it is actually possible to see the interference patterns and to refocus the shock-wave edges in PCDIH^35^. The origins of this effect, however, were not well understood. In this work, we construct a simulation environment to describe the possible sources of these visible features. The separation between objects and camera image planes are numerically simulated using the diffraction equation described in Eq. (1) and pure absorption was used to model the wires. Shock-wave simulation results illustrated in Fig. 7 incorporate a spherical phase distortion^11,57^ with a constant shocked-gas density^58–61^, shock-wave motion during laser time-of-flight, and light refraction effects at shock-wave edges. Additional simulations and parameter details are discussed in Supplementary Note 3.

These simulations display several features that are seen in experimental data. First, the DIH simulations and experimental data both show strong interference patterns near shock-wave edges (bright fringe directly inside the dark refocusable edge). Similarly, the PCDIH simulations and experiments both show weaker interference patterns. Second, the shock-wave edge is refocusable in simulation and in experiments for both DIH and PCDIH. While the refocusable shock-wave edge in DIH is due to the structure or size of the phase discontinuity, simulations further indicate that the refocusable shock-wave edge in PCDIH is due mostly to shock-wave motion during laser light time-of-flight. Similarly, fast temporal changes in index of refraction, density, gas ionization, or gas dissociation during the laser light time-of-flight will also produce visible artifacts in PCDIH images. Based on additional experiments with stationary supersonic shock-waves (see Supplementary Note 2 and Supplementary Fig. 1) and additional simulations (see Supplementary Figs. 2 and 3), it is also clear that light refraction from shock-wave curvature plays an important role. Finally, just like the experimental data in Fig. 4, the simulated shock-wave distorts the edges of the vertical wire in DIH but not PCDIH. These results show that this model captures most of the visible features and artifacts in both processes.

With a better understanding of these mechanisms, the ultra-high-speed PCDIH system is next applied for removing phase distortions from shock-waves created by hypersonic fragments generated using a small explosive bridgewire detonator, as illustrated in Fig. 1a. When the explosive ignites, fragments of the brass cap are launched into the field-of-view ahead of the main explosive fireball. The hypersonic fragments generate strong shock-waves as they travel, which obscures other fragments that are traveling slightly behind the leading fragments. In order to better understand and model explosive dynamics as well as secondary ignition events, it is important to track these fragments and capture the shock–wave interactions over time^62^.

**Fig. 8:**
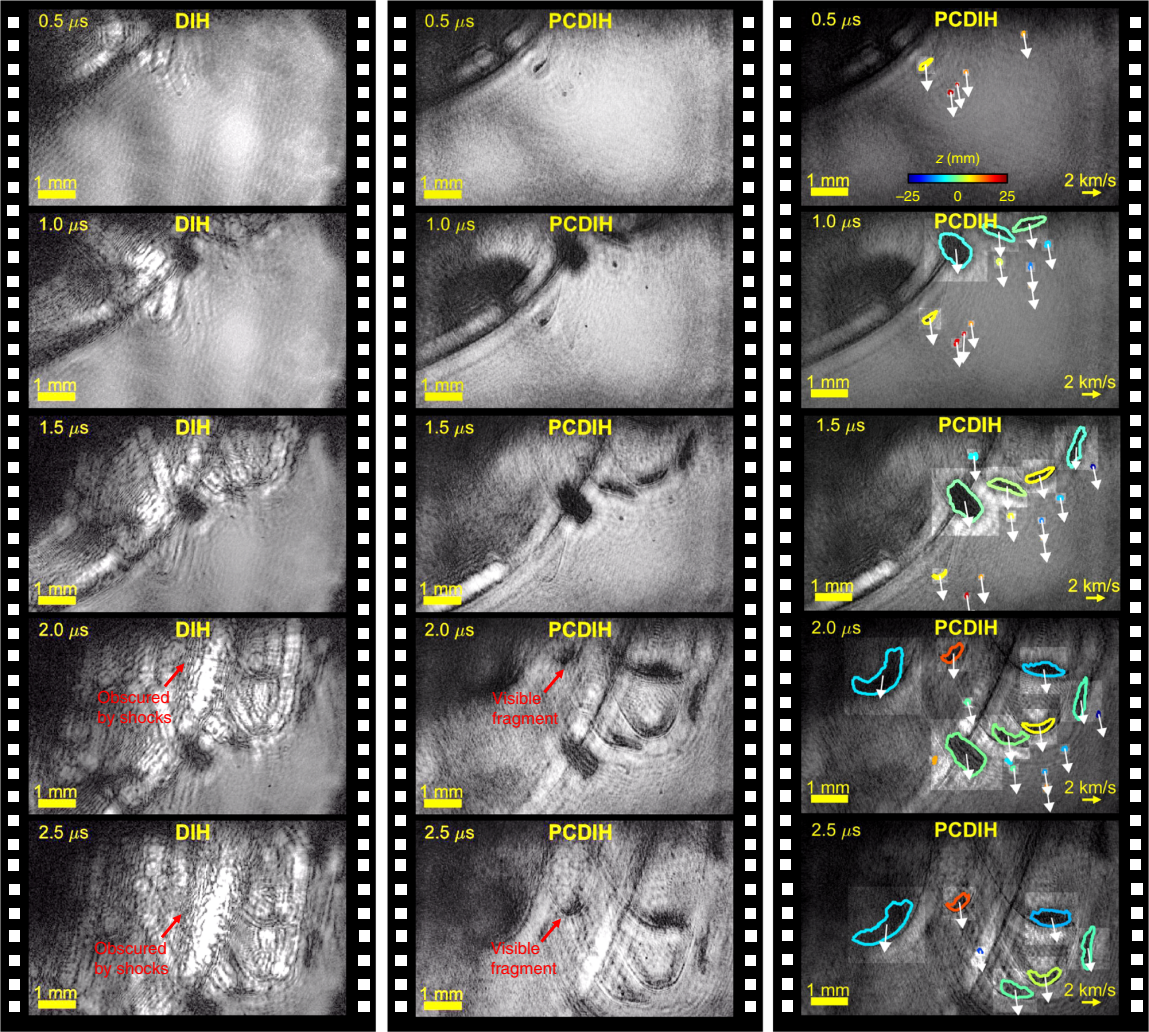
Video of explosively generated hypersonic fragments collected at 2 MHz. Columns from left to right show DIH, PCDIH, and tracked PCDIH videos of the hypersonic fragments and surrounding shock-wave distortions. Late-arriving fragments generated by an explosive detonator are obscured by shock-wave phase distortion interference patterns. However, the fragments are visible and can be tracked in the PCDIH images to estimate size, three-dimensional position (*z*-location illustrated by outline color), and velocity. All holograms are numerically refocused to the center of the fragment field. Supplementary Movie 3 illustrates this experiment.

A 2 MHz video illustrating the motion of the hypersonic fragments and shock-waves is illustrated in Fig. 8. The data are refocused to the center of the fragment field. The first frame shows the edge of one large fragment and several smaller sub-millimeter leading fragments. This is followed by a series of larger fragments that are clearly visible in PCDIH. The PCDIH holograms can then be used to numerically refocus each fragment such that their in-focus *z*-planes and outline shapes can be determined, as shown in the right-most column of Fig. 8 and in Supplementary Movie 3. These particles are then tracked over time to determine their velocities, which vary between 2.34 and 2.83 km s^−1^ (Mach 6.8 to Mach 8.2). This example clearly illustrates how the ultra-high-speed PCDIH technique can enable 3D object tracking in explosive environments using a single line of sight.

## Discussion

The ultra-high-speed PCDIH technique for imaging through shock-wave distortions demonstrates a new capability for holographic imaging and time-resolved 3D tracking in dynamic environments. Video rates of up to 5 million-frames-per-second were obtained using a custom-modified pulse-burst laser and ultra-high-speed cameras. Where previous work was limited to 10–20 Hz^35,36^, this work demonstrates a speed increase in excess of five orders of magnitude, enabling the study of ultra-fast, transient events including laser-spark plasma-generated shock-waves and explosively generated hypersonic fragments. The new dynamic data has enabled distortion-canceled shock-wave velocity measurements, fragment velocity measurements, object tracking, and 3D observations of shock-wave interactions, all of which are new capabilities.

In previous work^35^, the source of the weak interference patterns in PCDIH images were not well understood, hence the ability to predict success in other applications was limited. In this paper, we use physics-based simulations and experimental evidence from laser-spark plasma-generated shock-waves and stationary supersonic shock-waves to illustrate how shock-wave motion during laser light time-of-flight and refraction contribute to these features. These simulations also show that gas absorption at shock-wave edges do not play a role, as previously hypothesized^35^. These new simulations advance our understanding of how coherent phase conjugate light interacts with shock-waves and density gradients. With this model and an improved understanding of the physical processes, we can confidently extend the technique to other application areas. Although the remaining interference patterns in PCDIH images from these sources cannot be corrected optically, these mechanisms do enable refocusing of shock-wave edges, which opens up the potential for applications that require 3D tracking of shock-waves with minimized phase distortions.

Overall, the PCDIH technique significantly reduces bright interference patterns caused by phase distortions, enabling numerical refocusing of absorptive objects or scattering sources. This technique has a few disadvantages including increased setup complexity and higher laser pulse energy requirements. At the highest repetition rates, the signal-to-noise ratio of PCDIH can be low and plasma emissions or specular reflections may dominate. However, ultra-high-speed PCDIH shows drastic benefits over traditional DIH for time-resolved 3D object tracking in environments that are otherwise confounded by shock-wave distortions. The innovations outlined in this work advance how flashlamp-pumped pulse-burst lasers can be used in the future for both coherent imaging and four-wave-mixing applications. In addition to the examples presented in this paper, ultra-high-speed PCDIH can be applied for removing distortions due to shock-waves or ionized gases from test articles, particles, and liquid droplets in extreme supersonic and hypersonic shock-tube and wind-tunnel experiments. Even though this paper focuses on shock-waves, ultra-high-speed PCDIH may have profound impacts in other environments with strong phase distortions from turbulence, thermal gradients, immiscible fluid boundaries, bubble cavitation, gas ionization, and other sources.

## Methods

### PCDIH setup

The simplified experimental setup illustrated in Fig. 2 implements PCDIH using degenerate four-wave mixing. Alternative phase conjugate mirror designs, such as stimulated Brillouin scattering were also considered. However, due to laser breakdown inside the fluid cell, these methods were not implemented in the final design. Here, a custom modified Spectral Energies QuasiModo pulse-burst laser is used to generate MHz-rate pulses with pulse durations of  ~3 ns, burst durations of 0.5–1.5 ms, and inter-burst times of 12 s. This laser contains a pulsed seed source with a 2 GHz bandwidth, two double-pass diode-pumped stages, two double-pass 9 mm diameter flashlamp-pumped stages, and two single-pass 12 mm diameter flashlamp-pumped stages. The amplified 1064 nm light is compressed using a 4:1 telescope and doubled to 532 nm in a 30 mm long lithium triborate (LBO) crystal.

Next, the 532 nm light is sent through a two-stage attenuator and then split into an imaging beam and a single pump beam. Since nanosecond laser systems have long pulse durations and coherence lengths, this configuration was chosen to maximize pump energy, reduce the total number of components, and simplify alignment. The beams are delayed so that they arrive at the phase conjugate mirror nearly simultaneously. The imaging beam first passes through a vacuum pinhole spatial filter (300 mm FL lenses, 100 μm pinhole), a 3× beam expander, the PCDIH pickoff mirror (polarized beam splitter reflecting s-polarizations), the object area, and DIH pickoff mirror (5% reflectivity). Then, the imaging beam is focused (*f* = 125 mm lens) through a heavy fluorocarbon 3M FC-72^55^ fluid cell (AR coated 1 mm-thick windows, 10 mm-thick cell). The focal point of the imaging beam is placed slightly behind the cell to prevent breakdown inside the liquid or damage to windows.

The pump 1 beam passes through a telescope to control collimation, correct laser beam divergence, and increase energy density inside the cell. Pump 1 then passes through the phase conjugate mirror and quarter wave plate before reflecting off of a 90° mirror. The resulting beam then passes through the phase conjugate cell as pump 2 with the opposite polarization. The angle between the pump beams and the imaging beam is shallow at 23° to maximize beam overlap and increase phase conjugate mirror reflectivity.

The phase conjugate signal (dotted green lines) generated inside the cell then back-propagates along the imaging path, through the object area, and is reflected off the s-polarized pickoff mirror into the PCDIH camera. This new configuration helps reject p-polarized background and reflections. In summary, the pump 1, pump 2, imaging, and phase conjugate beams are p-, s-, p-, and s-polarized, respectively. The signal-to-noise ratio of the PCDIH beam is better than 60:1 when operating at ≤1 MHz. Neutral density filters are also added to adjust image intensity and laserline filters are used to minimize undesirable emissions from plasmas and explosives.

For ultra-high-speed imaging, Shimadzu HPV-X2 cameras are utilized. These cameras are capable of operating at up to 5 MHz at full frame (400 × 250 pixels, 10-bit depth, 32 μm pixel pitch, 128 frames max). Infinity K2 DistaMax long distance microscopes with STD or CF2 lenses are used for high magnification imaging at long stand-off distances. Since the events studied in this work are relatively short, this requires only a few frames to be captured per test and does not require pulse-to-pulse synchronization between the laser and cameras.

### Exemplary experiments

In the exemplary experiments, the laser-spark was generated using a Continuum Surelite III (10 Hz, 1064 nm, 5 ns pulse duration, 400 mJ/pulse) focused to a point using a 100 mm lens. For the explosive bridgewire detonator experiments, a polycarbonate boombox is used to contain the fragments and protect the optics outside the object area. A small RP-80 detonator from Teledyne RISI with an additional 0.18 mm-thick brass cap is oriented downward and positioned 50 mm above the field-of-view prior to detonation.

## Supplementary information

Supplementary Information File

Description of Additional Supplementary Files

Supplementary Movie 1

Supplementary Movie 2

Supplementary Movie 3

## Data Availability

The data for Fig. 3 and Supplementary Fig. 3 are provided in the Source Data file. Other datasets generated and/or analyzed during the current study are available from the corresponding author upon reasonable request.

## Source data

Source Data

## Code availability

The HoloSAND hologram refocusing and particle identification code is available by request from www.osti.gov under the OSTI identification code 1242887. Additional simulation and processing codes are available from the corresponding author upon reasonable request.
