# Supplementary material for: Megahertz-rate shock-wave distortion cancellation via phase conjugate digital in-line holography
Source: Nat Commun. 2020 Feb 28;11:1129. doi: 10.1038/s41467-020-14868-y (PMC7048751; doi:10.1038/s41467-020-14868-y)
Supplement: Supplementary file 1 — Supplementary Information File [file 41467_2020_14868_MOESM1_ESM.pdf]

Megahertz-rate Shock-wave Distortion Cancellation via Phase Conjugate Digital In-line Holography

Mazumdar et al.

Megahertz-rate Shock-wave Distortion Cancellation via Phase Conjugate Digital In-line Holography

Yi Chen Mazumdar[1,2*], Michael E. Smyser[3], Jeffery D. Heyborne[1], Mikhail N. Slipchenko[3], Daniel R. Guildenbecher[1]

[1]Sandia National Laboratories, P.O. Box 5800, Albuquerque, NM, 87185, USA.
[2]School of Mechanical Engineering, Georgia Institute of Technology, Atlanta, GA, 30332, USA.
[3]School of Mechanical Engineering, Purdue University, West Lafayette, IN, 47907, USA.
[*]Corresponding author: ellen.mazumdar@me.gatech.edu

Supplementary Information

Supplementary Note 1: Degenerate Four-wave Mixing

To create the phase conjugate signal, a degenerate four-wave-mixing topology is utilized.[53,54] In this scheme, a third-order nonlinear medium is pumped by two planar counter-propagating beams ($E_1 = A_1(r)e^{ik_1\cdot r-i\omega t}$ and $E_2 = A_2(r)e^{ik_2\cdot r-i\omega t}$) and a separate imaging beam traveling along the z-axis ($E_3 = E'_{\mathrm{t}} = A_3(x,y,z)e^{ik_3 z-i\omega t}$ and $k_3 = k$). The angle between the pump beams and the imaging beam is shallow to maximize overlap length l and improve phase conjugate signal intensity. When these three beams interact in the nonlinear medium, a fourth beam E_4 is generated. From phase matching conditions, $k_1 + k_2 = k_3 + k_4$, and counter-propagating pump beams, $k_1 = -k_2$, we can determine that the phase conjugate beam propagates in the opposite direction with respect to the image beam, $k_3 = -k_4$. The nonlinear polarization fields are then,

$$P_3^{(3)}(x,y,z) = \epsilon_0\chi_{\mathrm{e}}^{(3)}A_1A_2A_4^*e^{-ik_3z-i\omega t}, \tag{1}$$

$$P_4^{(3)}(x,y,z) = \epsilon_0\chi_{\mathrm{e}}^{(3)}A_1A_2A_3^*e^{ik_3z-i\omega t}, \tag{2}$$

where $\chi_{\mathrm{e}}^{(3)} = \chi^{(3)}(\omega,\omega,-\omega)$ is the effective third-order nonlinear susceptibility. Amplitude variations can be obtained by substituting the polarization fields into the nonlinear coupled-wave equations such that,[53]

$$\frac{dA_3^*(z)}{dz} = i\gamma^*A_4(z) \tag{3}$$

$$\frac{dA_4(z)}{dz} = i\gamma A_3^*(z) \tag{4}$$

$$\gamma = \frac{\omega}{2cn_0(\omega)}\chi_{\mathrm{e}}^{(3)}A_1A_2. \tag{5}$$

Assuming that the pump beam amplitudes stay constant, the magnitude of the phase conjugate signal evaluated at one face of the phase conjugate mirror at z_0 is,[53]

$$A_4(z_0) = -i\frac{\gamma}{|\gamma|}\tan(|\gamma|l)A_3^*(z_0). \tag{6}$$

From this equation, we see that A_4 is proportional to the conjugate of the imaging beam A_3. The resulting electric field of the phase conjugate wave assuming small signal gains is then $E_4(x,y,z) = E''_{\mathrm{t}}(x,y,z) = A_4(x,y,z)e^{ik_4z-i\omega t}$. Combining these equations, we can determine the phase conjugate mirror reflectivity,

$$R_{\mathrm{pc}} = |A_4(0)|^2/|A_3(0)|^2 = \tan^2(|\gamma|l) = \tan^2\left(\left|\frac{\omega}{2cn_0(\omega)}\chi_{\mathrm{e}}^{(3)}A_1A_2\right|l\right). \tag{7}$$

The reflectivity of the phase conjugate mirror depends heavily on the third-order nonlinear susceptibility, the interaction length, and the energy density of the two pump beams.

Supplementary Note 2: Stationary Supersonic Jets

In order to better understand the physical mechanisms that dominate in holographic imaging of shock-wave distortions, a stationary supersonic jet was studied. For these experiments, the setup was modified and a picosecond laser (Ekspla PL2231C-20, 20 Hz, 60 ps, 532 nm, >15 mJ per pulse), high resolution cameras (LaVision sCMOS, 2560 $\times$ 2160 pixels, 6.5 µm pixel pitch, 16-bit depth), and a separate pump 2 beam are used.[48] The air-driven stationary supersonic jet (over-expanded jet, design Mach number of 3.7, 6.35 mm nozzle outlet, stagnation pressure 4.3 MPa, atmospheric pressure 84 kPa for Albuquerque, New Mexico, USA) is illustrated in Supplementary Figure 1a. This jet is first studied with the focal planes of both cameras placed at the center of the nozzle, as illustrated in the left column of Supplementary Figure 1b. In this mode, images of the shock-waves are collected rather than holograms. The in-focus images from the DIH camera show sharp shock-wave edges due to light refraction and some turbulent disturbances. The PCDIH camera shows these same structures but has darker shadows. The darker shadows are likely due to additional absorption, refraction, and diffraction losses from the phase conjugate mirror and the second-pass of the light through the object area.

Next the imaging planes of both cameras are moved to a distance of ~97 mm from the centerline and in-line

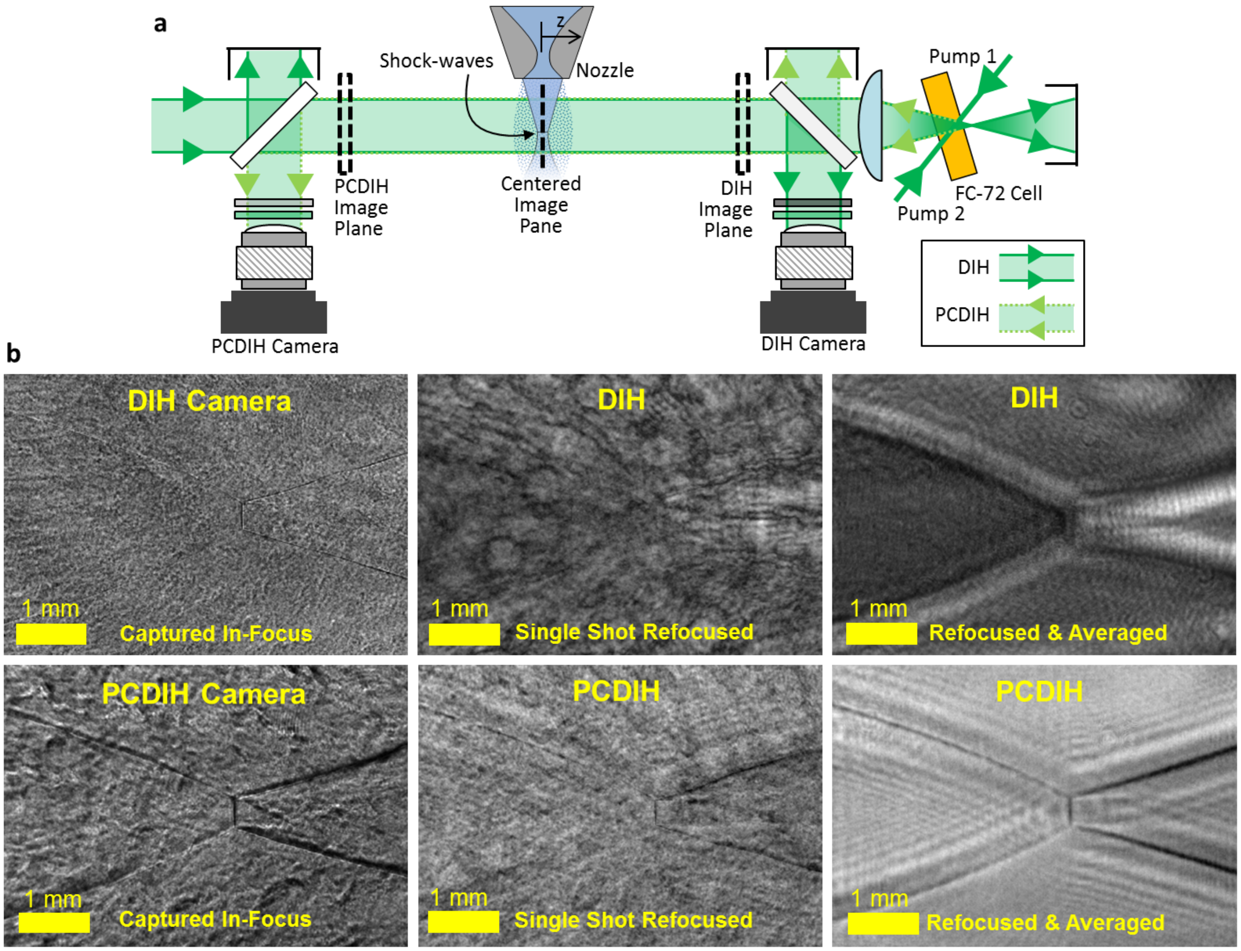

Supplementary Figure 1: DIH and PCDIH measurements of a stationary supersonic jet. **a**, A schematic of a stationary supersonic jet measured using a picosecond laser configuration is illustrated. **b**, The left column shows normal imaging of the jet captured in focus (focal planes at the centered image plane). Next, the focal planes are moved to the PCDIH and DIH image planes. The center column shows single-shot refocused holograms of the jet and the right column shows refocused and averaged (from 500 instances) images of the jet with turbulence effects minimized.

holograms are collected, as illustrated in the middle column of Supplementary Figure 1b. In these single-shot images, it was not possible to refocus to the shock-wave edges in DIH but was possible to refocus in PCDIH. The quality of the hologram images are slightly lower than the case with direct imaging partially due to the presence of the virtual image inherent to the DIH technique[50,51] and partially due to the presence of turbulent structures. When 500 images were collected and averaged, as illustrated in the right column of Supplementary Figure 1b, the turbulent disturbances were removed and the shock-wave edges are more clearly visible. Again, the shock-wave edges are refocusable in PCDIH but not in DIH.

This experiment illustrates several interesting features. In contrast to previous applications of PCDIH, the shock-waves imaged here are stationary. The results indicate that the visible edges in PCDIH imaging cannot be explained solely by shock-wave motion during the laser beam time-of-flight. Absorption in the high density regions near the shock-wave edges is also an unlikely contributor due to the short path lengths. Rather, refractive losses caused by the sharp index-of-refraction gradients at the shock-wave edges appear to be the more likely source. This is studied further via simulations presented in the next section. Finally, phase distortions make it difficult to refocus to shock-wave edges in DIH because they dominate over interference patterns generated by refraction. Therefore, PCDIH has a distinct advantage over DIH for both distortion cancellation and shock-wave edge refocusing.

Supplementary Note 3: Simulations of Shock-wave Interactions

Simulating the detailed mechanisms that drive the image formation process in DIH and PCDIH is important for understanding the diffraction pattern features seen in both methods. Several different models can be utilized to simulate phase distortions,[11,57] but for the laser-spark plasma-generated shock-wave example, where the shock-wave and absorptive objects are in distinctly separate planes, simplified simulation mechanisms can be used.

For this work, higher spatial resolutions are simulated and then down-sampled at the DIH and PCDIH image planes to match the spatial resolution of the Shimadzu ultra-high-speed cameras. The wires are simulated as pure planar absorptive objects $a(x, y)$ and the shock-waves are simulated as planar objects that are a combination of phase distortions $\phi(x, y)$, refraction, and absorption. The propagation between objects and camera image planes are simulated using the diffraction equation described in Eq.(1). The DIH simulation places the vertical wire plane at $z = -10$ mm, shock-wave plane at $z = 0$ mm, and horizontal wire plane at $z = 10$ mm with the DIH image plane placed further downstream at $z = 30$ mm. The PCDIH simulation places a phase conjugate mirror ($E''_\mathrm{t}(x, y, z) = R_\mathrm{pc} E'^*_\mathrm{t}(x, y, z)$) 500 mm downstream from the horizontal wire. The light then propagates back upstream through the horizontal wire, shock-wave, and vertical wire before entering the PCDIH image plane at $z = -30$ mm. At the image planes, noise is added and then the holograms are numerically refocused using Eq.(2).

Simulation result are illustrated in Supplementary Fig. 2. First, an idea spherical ($2R$ =1.2 mm) laser-spark plasma-generated shock-wave is simulated with a constant internal shocked-gas density ($n_\mathrm{gas} = 1$ and $n_\mathrm{air} = 1.0002$ at an atmospheric pressure of 84 kPa). The phase delay of the shock-wave is simulated by integrating through the spherical shape[57] such that $\phi(x, y) \approx 2\pi R(n_\mathrm{gas} - n_\mathrm{air})/\lambda\sqrt{1 - z^2/R^2}$. In the example from the first column, there is no shock-wave motion, misalignment, absorption, or refraction so the phase distortion perfectly cancels on the second pass. Therefore, the PCDIH simulation shows no shock-wave. The DIH image, however, shows a bright fringe directly inside the shock-wave edge. The basic structure of the interference pattern matches experimental results in Fig. 4 of the main paper.

A more complex density distribution model following Sedov-Taylor blast-wave theory[60,61] can also be simulated. After solving the Sedov-Taylor equations for the density distribution as a function of radius, as shown in Supplementary Fig. 3, the function can be numerically integrated as a function of z through the shock-wave to generate the phase delay $\phi(x, y)$. This example is illustrated in the second column of Supplementary Figure 2. The shock-wave in this case is refocused to a white edge and a dark internal gradient near the edge. Brighter external fringes are also visible. These features, however, are not observed in experimental data. At early times, as illustrated in Fig. 5 from the main paper, the laser-spark is approximately the same size as the shock-wave and the shock-wave has yet to coalesce into a sphere, which makes this system different from the assumptions for the Sedov-Taylor model. The true density distribution is complex and somewhere between the Sedov-Taylor model and a constant density model.[58,59] Overall, we observe that the constant shocked-gas density model better approximates the measured holography data.

Since a pure phase-delay would predict no shock-wave distortion signal for PCDIH, as shown in the left two columns, additional mechanisms need to be added to the model. In the next column of Supplementary Fig. 2, the size of the shock-wave is increased slightly on the second pass of the laser beam to simulate shock-wave motion

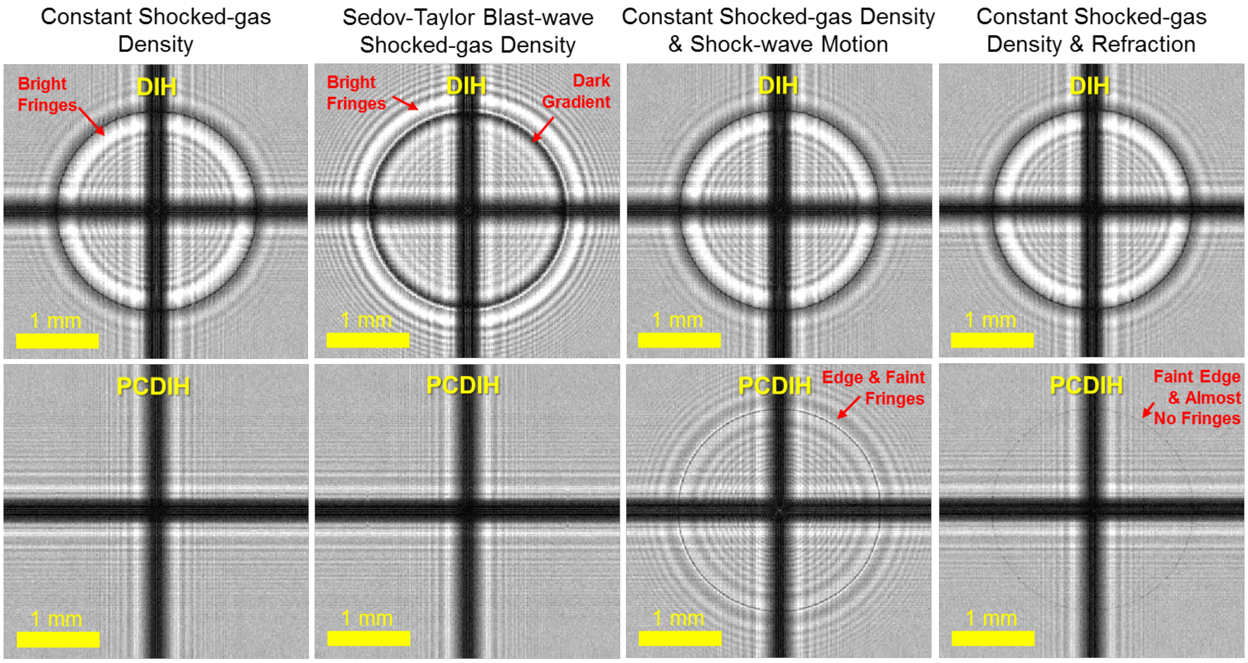

Supplementary Figure 2: Simulations of optical mechanisms contributing to DIH and PCDIH signals. The columns from left to right simulate a constant shocked-gas density, a Sedov-Taylor blast-wave shocked-gas density distribution, a constant shocked-gas density with shock-wave motion during the laser light time-of-flight, and a constant shocked-gas density with light refraction at the shock-wave edges. These simulations are refocused to the shock-wave focal plane.

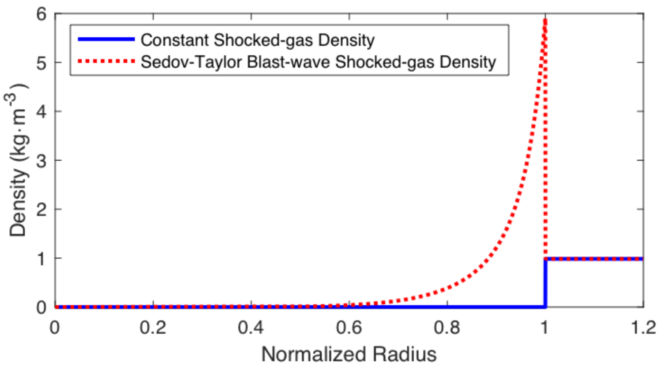

Supplementary Figure 3: Simulated density distributions as a function of normalized radius. A constant shocked-gas density and a Sedov-Taylor blast-wave shocked-gas density are compared as a function of normalized radius along the centerline of the spherical shock-wave. Simulation source data are provided in the Source Data file.

during laser light time-of-flight. For a shock-wave to phase conjugate mirror to shock-wave distance of 1020 mm, a measured shock-wave speed of $\geq$ 2 km·s^{-1}, and a laser pulse-length of $\sim$3 ns, the shock-wave is expected to travel between 6.8 to 12.8 µm during laser light time-of-flight. With this additional effect, a refocusable shock-wave edge can be measured in the PCDIH image, with maximum shock-wave edge sharpness measured within 1 mm of $z = 0$. This small focus offset is likely because the phase conjugate hologram is generated from coherent interference of the forward and reverse phase distortions and not from a real absorptive object. These PCDIH simulation results show many similarities to the experimental data. It is important to note that even small misalignments and beam divergence would create similar effects on the PCDIH signal and are therefore also possible sources of the visible shock-wave interference patterns in PCDIH.

Lastly, based on stationary supersonic jet imaging results, refraction at shock-wave edges is included in the model. Refraction in this case is modeled using the Fresnel equations for transmitted power when entering (T_1) and when exiting (T_2) the spherical shock-wave assuming that the path of light is not significantly shifted due to the small difference between n_{gas} and n_{air},

$$T_1 = 1 - \left| \frac{n_{\text{air}} cos\theta_{\text{i}} - n_{\text{gas}} cos\theta_{\text{t}}}{n_{\text{air}} cos\theta_{\text{i}} + n_{\text{gas}} cos\theta_{\text{t}}} \right|^2 \text{, for } \theta_{\text{i}} = sin^{-1}\left(\frac{\sqrt{x^2+y^2}}{R}\right) \text{ and } \theta_{\text{t}} = sin^{-1}\left(\frac{n_{\text{air}}}{n_{\text{gas}}} sin\theta_{\text{i}}\right), \tag{8}$$

$$T_2 = 1 - \left| \frac{n_{\text{gas}} cos\theta_{\text{i}} - n_{\text{air}} cos\theta_{\text{t}}}{n_{\text{gas}} cos\theta_{\text{i}} + n_{\text{air}} cos\theta_{\text{t}}} \right|^2 \text{, for } \theta_{\text{i}} = sin^{-1}\left(\frac{\sqrt{x^2+y^2}}{R}\right) \text{ and } \theta_{\text{t}} = sin^{-1}\left(\frac{n_{\text{gas}}}{n_{\text{air}}} sin\theta_{\text{i}}\right). \tag{9}$$

This is then combined with the Beer-Lambert law to estimate apparent absorption $\alpha \approx log_{10}(1/(T_1 T_2))$ due to light refracted out of the imaging volume. More complex Debye series models can also be utilized to achieve this effect.[11, 57] With this model, refractive losses only occur at the shock-wave edges due to the local curvature, making faint edges visible in PCDIH when refocused. When compared with experimental data, these simulation results indicate that a model incorporating constant shocked-gas density, shock-wave motion, and refraction captures most of the visible distinguishing features of DIH and PCDIH.

For both DIH and PCDIH simulations of the wire, the uncertainty in the z-depth location based on the focus metric is 0.5 to 1 mm, which is similar to estimates from other experiments.[5, 52] For DIH simulations with no shock-wave distortions and PCDIH simulations with cancelled shock-wave distortions, the measured z-location of the wire is at the original simulated location. However, in areas where the wire is not completely obscured, the DIH simulations with a shock-wave distortion show that the in-focus location of the wire is offset or biased by 1.5 to 2 mm from the original simulated location. These results indicate that the accuracy of the z-location estimate that is lost by the introduction of a shock-wave distortion is later recovered by the PCDIH phase distortion cancellation process.
