# Supplementary material for: Megahertz-rate shock-wave distortion cancellation via phase conjugate digital in-line holography
Source: Nat Commun. 2020 Feb 28;11:1129. doi: 10.1038/s41467-020-14868-y (PMC7048751; doi:10.1038/s41467-020-14868-y)
Supplement: Supplementary file 2 — Description of Additional Supplementary Files [file 41467_2020_14868_MOESM2_ESM.pdf]

Description of Additional Supplementary Files

File Name: Supplementary Movie 1 : Numerical refocusing of a DIH and a PCDIH hologram to the focal planes of the vertical wire, shock-wave edge, and horizontal wire

Description: Two holograms are obtained using the DIH technique and the PCDIH technique. These two images are numerically refocused to different z-locations to show the in-focus vertical wire, laser-spark plasma-generated shock-wave edges, and horizontal wire. PCDIH images show significantly reduced distortions and no bright fringes. This movie is associated with the data in Fig.

File Name: Supplementary Movie 2 : Laser-spark plasma-generated shock-wave evolution over time captured via DIH and PCDIH at 500 kHz and 5 MHz

Description: A laser-spark plasma-generated shockwave between a pair of crossed wires is visualized. At the lower repetition rate of 500 kHz, the shock-wave is only visible on one frame, making it difficult to determine dynamics. At 5 MHz, the dynamics of the shock-wave expansion are visible. The plasma emission from the laser-spark compete with the lower PCDIH signals at the highest frame rate. The first half of this movie at 500 kHz is associated with Fig. 4 and the second half of this movie at 5 MHz is associated with Fig. 5.

File Name: Supplementary Movie 3 : Explosively-generated hypersonic fragments captured with DIH and PCDIH imaging techniques at 2 MHz

Description: The shock-waves from hypersonic fragments generated with an explosive bridgewire detonator are imaged with DIH and PCDIH. Tracking algorithms are then used to determine the z-location and velocity of fragments in the PCDIH images. This movie is associated with the data in Fig. 8.
